# A Case of Immunoglobulin G4-Related Ophthalmic Disease With Unilateral Visual Field Impairment

**DOI:** 10.7759/cureus.27495

**Published:** 2022-07-31

**Authors:** Shintaro Kohno, Hitoshi Tabuchi, Atsuki Fukushima

**Affiliations:** 1 Ophthalmology, Tsukazaki Hospital, Himeji, JPN; 2 Ophthalmology, Hiroshima University, Hiroshima, JPN

**Keywords:** igg4, immunoglobulin g4, visual field, visual disturbance, steroid use, pituitary gland, igg4-related disease

## Abstract

A 76-year-old man receiving maintenance therapy with oral steroids for immunoglobulin G4 (IgG4)-related disease presented to our hospital with the chief complaint of visual disturbance. His best corrected visual acuities of the right and left eye were 1.2 and 0.7, respectively. Humphrey visual field test revealed inferior auriculotemporal one-quarter blindness in the left eye. After detailed history-taking for IgG4-related disease, clinical diagnosis based on imaging revealed the marked pituitary/pituitary stalk enlargement with associated optic chiasm compression. Based on the history and initial evaluation findings, a diagnosis of IgG4-related ophthalmic disease was made. Intensified steroid therapy was performed, which led to symptom resolution. IgG4-related diseases are considered in the differential diagnosis when bilateral hemianopsia is observed. When unilateral visual acuity and visual field defects are present, IgG4-related diseases and other organ disorders should be considered.

## Introduction

Immunoglobulin G4 (IgG4)-related disease is an autoimmune disease of unknown etiology in which IgG4-positive plasma cells infiltrate the body organs and cause swelling, nodules, and hypertrophic lesions in target organs [1]. IgG4-related disease can occur in any organ. Regarding ophthalmology, the orbit is most commonly disturbed [2]. Approximately 20% and 5% of patients have vision loss and visual field defects, respectively [3].

Two major pathological conditions can cause optic neuropathy in this disease: compression or encasement of the optic nerve by an enlarged tissue in the orbit and compression of the optic chiasm by the enlarged pituitary gland. In the former case, compression or encasement of the optic nerve by the enlarged extraocular muscle or localized or diffuse tissue proliferation in the orbit has been reported [4]. The latter is a condition that can develop in any disease that causes pituitary enlargement; however, few studies have reported such cases [5,6] and there are no case reports of visual impairment alone.

## Case presentation

A 76-year-old man visited his family physician at the end of April 2021 due to a visual disturbance in the left eye and was referred to our department on May 27, 2021. Ophthalmological history included bilateral cataract surgery, left ptosis (both in 2017), intrascleral fixation of left intraocular lens dislocation (in 2020), Irvine-Gass syndrome, and ocular hypertension. Detailed history-taking revealed that the patient received treatment for IgG4-related disease (retroperitoneal fibrosis; maintenance dose of prednisolone 2.5 mg/day) from his primary care physician.

**Figure 1:**
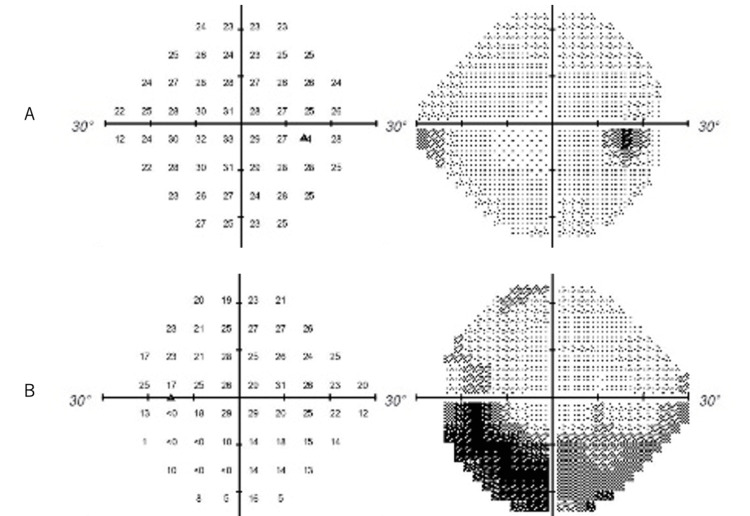
Humphrey visual field test on June 2. A: Right eye. No abnormal findings. B: Left eye. Note the inferior auriculotemporal 1/4 blindness in the left eye.

At the initial visit on May 27, 2021, there was no conjunctival hyperemia in both conjunctivae, and anterior chamber cells were occasional in the left eye. Pupillary reflexes were not disturbed and relative afferent pupillary defect (RAPD) was not noted in the left eye. The left optic nerve papilla was mildly erythematous and swollen. Best corrected visual acuities were 1.2 in the right eye and 0.7 in the left eye, and intraocular pressures were 18 mmHg in the right eye and 16 mmHg in the left eye. Critical flicker frequency (CFF) was decreased in the left eye (right ↑38Hz↓41Hz, left ↑36Hz↓36Hz), and Humphrey visual field test revealed inferior arcuate quadrantanopia in the left eye (Figure 1).

**Figure 2:**
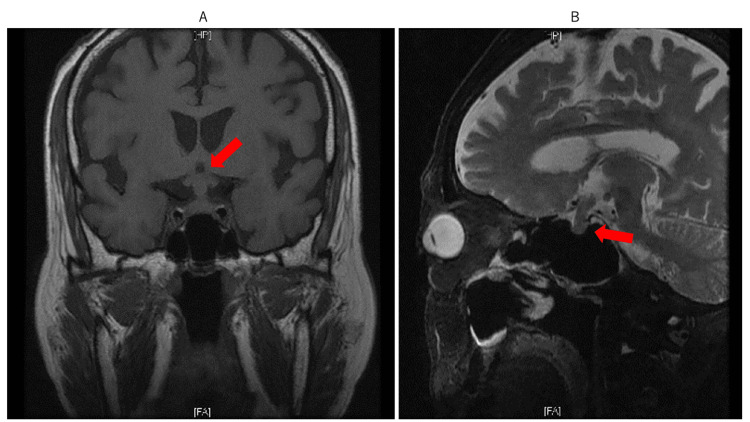
Brain MRI on June 2. A: Coronal section. B: Sagittal section. Note the marked pituitary/pituitary sclerenchyma enlargement (arrows) with associated optic chiasm compression without any abnormal findings in the optic nerve.

Brain magnetic resonance imaging (MRI) showed no abnormal signal in the intraorbital optic nerve; however, marked pituitary/pituitary stalk enlargement with associated optic chiasm compression was observed (Figure 2).

**Figure 3:**
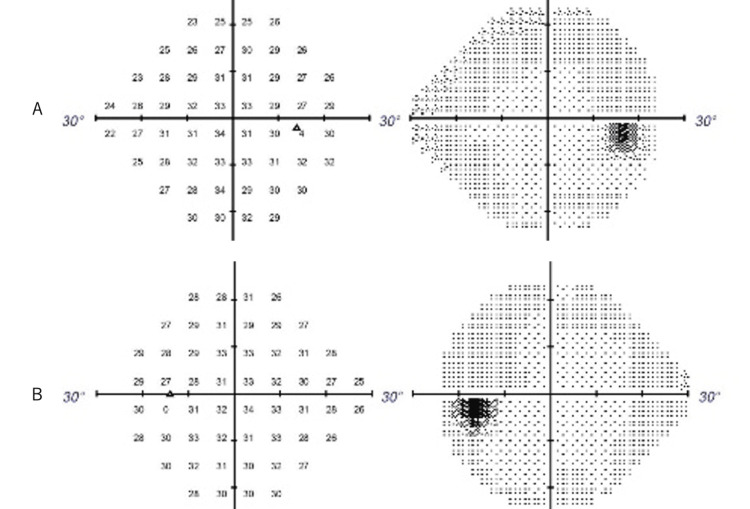
Humphrey visual field test on June 2. A: Right eye. B: Left eye. Note that abnormal findings in the left eye disappeared.

Based on the history and initial evaluation findings, a diagnosis of left optic neuropathy associated with pituitary/pituitary stalk enlargement caused by IgG4-related disease was made. He was introduced to the hospital of endocrinology and metabolism internal medicine expert and was diagnosed with panhypopituitarism due to pituitary funnel inflammation. Then, the dose of prednisolone was increased to 20 mg/day. On June 10, visual acuity and visual field defects improved (Figure 3).

**Figure 4:**
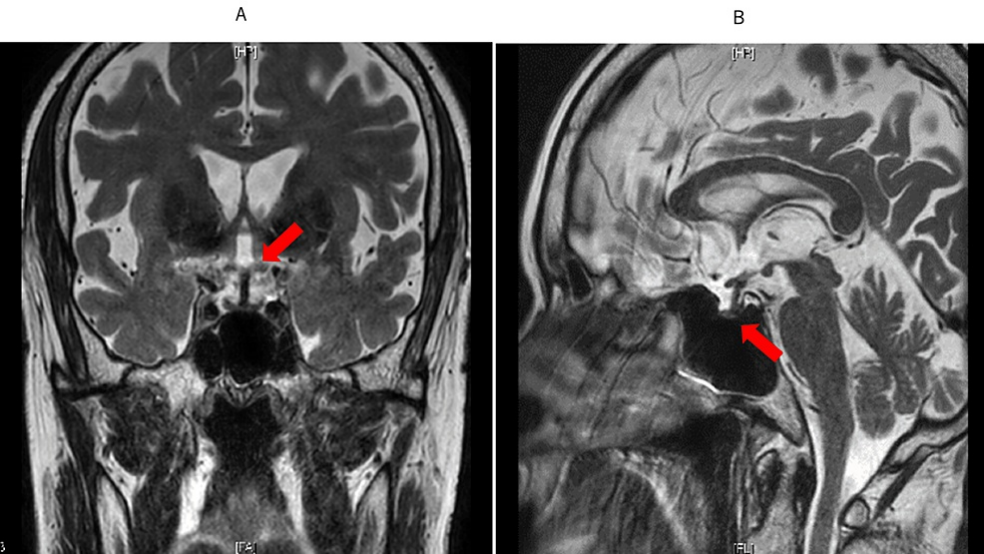
Brain MRI on June 2. A: Coronal section. B: Sagittal section. Note that the pituitary gland and pituitary pattern (arrows) were almost normalized.

Upon examination on July 7, the size of the pituitary gland and pituitary stalk decreased (Figure 4).

The CFF decreased (right ↑42Hz↓43Hz, left ↑37Hz↓43Hz) (39 Hz). The steroid dose was gradually decreased, and maintenance therapy with 2.5-mg prednisolone daily was resumed on August 11.

## Discussion

In this case, no optic nerve swelling or signal changes were evident on imaging, but fundus examination revealed mild erythema of the optic nerve papilla. Although this finding may be attributed to inflammatory cytokine production in the eye by iritis, optic neuritis was likely to be caused by an autoimmune mechanism in IgG4-related disease. However, the visual field findings were consistent neither with those commonly observed in optic papillitis nor pituitary enlargement. Moreover, brain MRI findings suggested optic chiasm compression due to pituitary enlargement. However, judging from our MRI data, the relationship of the optic chiasma to the pituitary gland was unclear and remained to be elucidated. The patient’s visual acuity and visual field defects improved after steroid treatment, suggesting that enlargement of the pituitary gland and pituitary stalk was the main pathophysiology of vision loss and visual field defects.

The ophthalmologic clinical hallmark of IgG4-related ocular disease is bilateral lacrimal gland enlargement with three features: suborbital nerve enlargement [7], exophthalmos, and compressive optic neuropathy [4]. IgG4-related optic neuropathy is commonly caused by compression of the optic nerve by enlargement of the lacrimal glands or other intraorbital tissues [4,8-11]. Few studies have reported on optic neuropathy caused by compression of the optic chiasm by an enlarged pituitary gland, as in the present case [5,10,11]. Although bitemporal hemianopsia is more commonly observed with pituitary enlargement, the present case showed inferior arcuate quadrantanopia only in the left eye. The exact mechanism of this visual field impairment remains unclear, as described above. A previous study [12] found visual field defects in only one eye in two of 10 patients with symmetrical pituitary enlargement. Because the present case also showed optic chiasm compression due to pituitary enlargement, it is possible that it caused an atypical visual field defect and associated visual acuity impairment.

Alternatively, as other studies have shown infiltration of IgG4-positive plasma cells around the trigeminal nerve in the same disease [4], a deep infiltrative or inflammatory mechanism may be involved in this case of optic neuropathy.

## Conclusions

IgG4-related disease, an autoimmune disease of unknown etiology, leads to vision loss and visual field defects. Compression or encasement of the optic nerve by an enlarged tissue in the orbit is reported to be the major cause of visual disturbance. However, the possibility of optic chiasm compression by the enlarged pituitary gland should be kept in mind.
